# Vitamin D status and VDR gene polymorphisms in patients with growth hormone deficiency: A case control Tunisian study

**DOI:** 10.1016/j.heliyon.2024.e34947

**Published:** 2024-07-20

**Authors:** Sarra Tombari, Yessine Amri, Yosra Hasni, Sondess Hadj Fredj, Yesmine Salem, Salima Ferchichi, Leila Essaddam, Taieb Messaoud, Rym Dabboubi

**Affiliations:** a Biochemistry Laboratory (LR00SP03), Bechir Hamza Children's Hospital, Tunis, Tunisia; b University of Jendouba, Higher Institute of Applied Studies in Humanity Le Kef, Department of Educational Sciences, Kef, Tunisia; c Department of Endocrinology, Farhat Hached Hospital, Sousse, Tunisia; d Biochemistry Laboratory, Farhat Hached Hospital, Sousse, Tunisia; e Department of Pediatrics, Bechir Hamza Children's Hospital, Tunis, Tunisia

**Keywords:** VDR gene polymorphisms, Vitamin D status, Haplotypes, Growth hormone deficiency

## Abstract

**Introduction:**

Growth Hormone Deficiency (GHD) is a rare disease marked by a complete or partial reduction in the production of growth hormone. Vitamin D deficiency is frequent and may be associated with several pathologies. However, the association between GHD and vitamin D deficiency has not been extensively studied. This study aimed to analyse VDR gene polymorphisms related to vitamin D status to ensure better care for patients with GHD.

**Material and methods:**

A case-control study was conducted at the Children's Hospital of Tunis in collaboration with the Farhat Hached's Hospital of Sousse, including patients with GHD and healthy subjects. Genetic analysis of the VDR gene polymorphisms was performed using PCR-RFLP technique. Haplotypes were examined with Haploview software, while statistical analyses were carried out using SPSS and R programming language.

**Results:**

Our study revealed significant differences in vitamin D (p = 0, 049) and calcium concentrations between patients and healthy subjects, which were lower in the GHD group (p = 0,018). A comparison of allelic and genotypic frequencies of the five polymorphisms indicated an association between the *Fok*I polymorphism and GHD. Furthermore, significant difference was observed between the *Apa*I genotypes and PTH (p = 0,019) and ALP (p = 0,035). *Fok*I genotypes were associated with phosphorus (p = 0,021). Additionally, One haplotype, CTAGT, exhibited a significant difference between the patients and healthy subjects (p = 0,002).

**Conclusion:**

Our study findings indicate that hypovitaminosis D is common among patients with GHD, even when undergoing treatment with rhGH. This underscores the critical importance of vitamin D supplementation during treatment.

## Highlights

• The association between GHD and vitamin D deficiency.
• Analyse of the interaction between VDR gene polymorphisms and vitamin D status.
• A positive association between the *Fok*I polymorphism and GHD.
• A significant difference between the *Apa*I genotypes and both PTH and ALP levels.
• The haplotype CTAGT was associated with GHD.

## 1 Introduction

Growth Hormone Deficiency (GHD) is a multigenic pathology considered as rare but treatable cause of short stature. A defect in the excretion of growth hormone (GH) leads to an alteration in longitudinal growth and various metabolic functions [1,2]. Growth, body composition, metabolism, bone mineral density, and quality of life are all affected by GH.

According to recent reviews, vitamin D deficiency is a condition whose prevalence varies widely from country to country, in some cases peaking at 98 % of the population in the population [[3], [4], [5]]. An estimated one billion people suffer from vitamin D deficiency, with a predominance of paediatric patients [6,7].

While vitamin D deficiency is the most common nutritional deficiency and probably the most common medical condition worldwide, it remains undiagnosed for a long time. In recent years, an increasing number of scientific studies have analysed the effects of vitamin D on the human body and have focused on the consequences of its deficiency, especially in paediatrics [8]. Indeed, vitamin D plays a pivotal role in regulating the metabolism of calcium and phosphorus, thereby influencing bone growth and mineralization processes significantly.

To date, more than forty genes have been incriminated in somatotropic axis defects, GHD being a multigenic abnormality of this axis, may be associated with other pathologies such as vitamin D deficiency. Hence, we postulated that investigating the impact of polymorphisms in the vitamin D receptor gene on GHD would be beneficial.

Numerous studies have focused on the evaluation of SNPs in the VDR gene, including the *Fok*I (rs2228570), *Bsm*I (rs1544410), *Apa*I (rs7975232), *Tru*9I (rs757343), and *Taq*I (rs731236) polymorphisms, and their association with vitamin D status.

Our study aims to examine the allelic polymorphism of the VDR in relation to the vitamin D status to ensure a better management of the GHD.

Our objectives are to establish firstly, the vitamin D status of GH-deficient patients compared to controls and secondly the phosphocalcic status of GH-deficient patients also compared to controls. We proposed to define the allelic polymorphisms of five distinctive regions of the VDR gene: *Taq*I (rs731236), *Apa*I (rs7975232), *Tru*9I (rs757343), *Bsm*I (rs1544410), and *Fok*I (rs2228570). Finally, we try to investigate the relationship between phosphocalcic balance parameters (Vitamin D, calcium, phosphorus, ALP, and PTH) and VDR gene polymorphisms.

## 2 Materials and methods

### 2.1 Subjects

A case-control study was conducted from March 2022 to May 2022 at Children's Hospital of Tunis in collaboration with the endocrinology department of Farhat Hached's Hospital of Sousse.

This study enrolled two groups of children: 39 subjects with GHD and a control group of 48 sex- and age-matched healthy children (control group).

The main criteria used for the selection of GHD patients were an age under 18 years old, a proven somatotroph deficiency by a stimulation test (GH < 10 ng/mL), a normal mental development and absence of chromosomic anomalies. The exclusion criteria were diagnosis of Turner syndrome or other syndrome affecting growth such as Russel Silver.

Both GHD Patients and healthy subjects should not have any chronic pathology or endocranial problems that affect growth, acute or chronic pathology that interferes with vitamin D metabolism (digestive, hepatic, or renal pathologies), treatment interfering with vitamin D and phospho-calcic metabolism, or treatments based on vitamin D or calcium three months prior to inclusion.

The Institutional Ethics Committee examined and approved the study protocol (Approval Number: 05/2023). Written informed consent was taken from all the parents and/or participants enrolled in the study.

### 2.2 Biochemical parameters

Samples of whole blood and plasma were collected after a minimum 12-h fast. Vitamin D levels were measured using chemiluminescence assay on a Mindray CL 1200i analyzer. The following table shows the quantification of other biological parameters (Table 1).

**Table 1:** Biological parameter assay methods.

Parameter	Method	Machine	Standard values
ALP	Enzyme colorimetry method	Cobas 6000	90–600 UI/L
PTH	Electrochemiluminescence	Cobas e411	15–65 pg/mL
Total Vitamin D	Chemiluminescence	Mindray CL – 1200i	30–80 ng/mL
Calcemia	Photometric method (Ca Gen2) Roche NM-BAPTA then EDTA	Cobas 6000	2.20–2.70 mmol/L
Phosphorus	Ammonium molybdate photometric method	Cobas 6000	1.1–2 mmol/L
Protidemia	Colorimetric method	Cobas 6000	60–80 g/L
Cholesterolemia	Cholesterol oxidase enzymatic method	Cobas 6000	3–6.2 mmol/L
Triglyceridemia	Colorimetric method	Cobas 6000	0.4–2.0 mmol/L

### 2.3 Genetic analysis

Using the PureLink®genomic DNA extraction kit (Thermofisher scientific), genomic DNA was extracted from blood samples that had been treated with EDTA. DNA quantification was performed using nanodrop at 260 nm.

The VDR gene's five polymorphisms were genotyped using the PCR-RFLP technique. Primers of five regions of VDR gene were obtained from the previously study of Guesmi A et al. [9] and verified using UCSC Genome Browser and Premier BioSoft (Table S1). A total of 25 μL was used for the PCR reaction, which included 500 ng of DNA, 0.3 mM of dNTPs, 3 mM of MgCl2, 0.5 U of Taq DNA polymerase, 1X buffer, 5–10 % DMSO, and 0.02 μM of each primer (forward and reverse). Restriction enzymes were used to digest PCR products overnight after amplification. The digested products were separated by electrophoresis on 2 % agarose gel.

In this study, the genotype of the genomic DNA samples was firstly determined using an ABI Prism 310 DNA sequencer (Applied Biosystems, Foster City, CA, USA) and then used as internal reference controls during PCR-RFLP method. Furthermore, to ensure the accuracy of our genotyping using PCR-RFLP, more than 10 % of the available DNA samples from both patients and controls were randomly selected for direct sequencing. The gel image of electrophoresed cut-and-digested PCR products is provided in Figs. S1–S5.

### 2.4 Statistical analysis

SPSS version 26 and R Studio software were used for statistical analysis. Results were considered statistically significant when the p-value was <0.05.

The Kolmogorov-Smirnov test was performed to identify Gaussian and non-Gaussian distributions. Student's t-test for independent samples was performed to compare quantitative variables if the distribution was normal (Gaussian), and Pearson's test was used to determine correlations. If the distribution was not Gaussian, the Spearman test was used for correlation and the Mann-Whitney *U* test was conducted for comparison. Both the parametric ANOVA test and nonparametric Kruskal-Wallis test were employed to compare more than two groups.

Qualitative variables were compared using Khi 2 test or exact fisher test for independent samples.

Haplotype analyses for the five polymorphisms were assessed using the Haploview 4.2 Software. The EM algorithm (Expectation-Maximization) was used to quantify the frequencies, and the Solid Spine of the LD algorithm was employed to ascertain the LD block structure. The test χ2 was used to estimate statistical differences in the distributions of allele and haplotype frequencies between case and control individuals.

## 3 Results

### 3.1 Patients’ characteristics

**Fig. 1:**
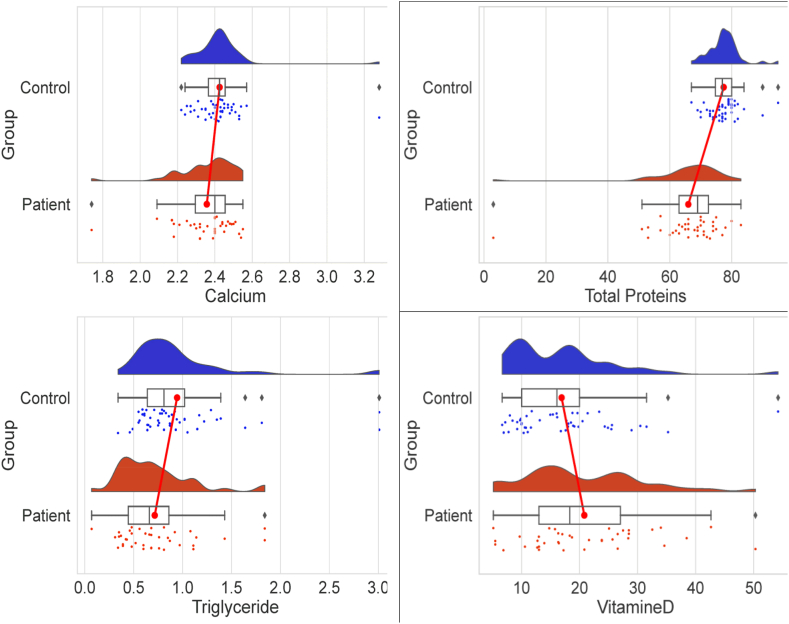
A descriptive analysis of the biochemical parameters in GHD patients and controls.

**Fig. 2:**
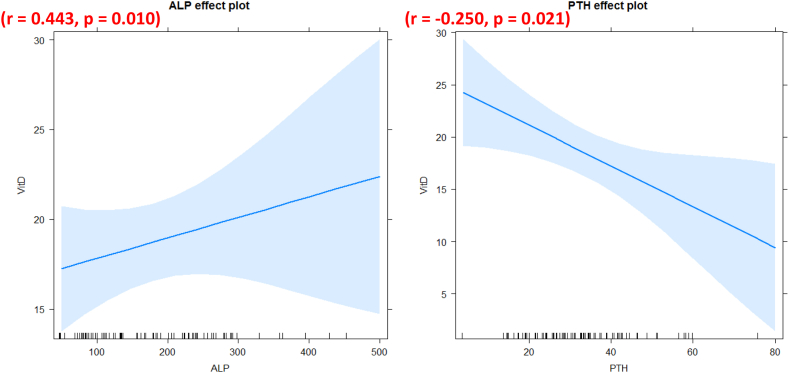
Correlation between vitamin D and phosphocalcic parameters (ALP left panel and PTH right panel).

Comparing GHD patients' biochemical and clinical characteristics with those of healthy controls showed notable differences in body weight (p = 0.003), height (p = 0.0001), total protein (p < 0.0001), triglycerides (p = 0.012), 25 (OH)D (p = 0.049) and calcium (p = 0.043). However, there were no differences in BMI (p = 0.59), total cholesterol (p = 0.48), PTH (p = 0.15), ALP (p = 0.65), or phosphorus (p = 0.62) (Table 2 and Fig. 1). In addition, statistical analysis revealed a negative correlation between PTH and vitamin D levels (r = −0.250, p = 0.021) and a positive one between ALP and vitamin D (r = 0.443, p = 0.010) (Fig. 2).

**Table 2:** Characteristics of the studied population.

Characteristics	Patients (n = 39)	Controls (n = 48)	p
Age (years)	14,03 ± 4997	14,29 ± 3848	0,91
Sex ratio	1,2 (56 % Males and 44 % Females)	1 (50 % Males and 50 % Females)	0,55
BMI (Kg/m^2^)	19,66 ± 4,76	19,94 ± 4,59	0,59
Total Proteins (g/L)	68 [2,8,9,9–82]	77 [67–95]	**<0,0001**
Total Cholesterol (mmol/L)	3,7 [0,39–6,45]	3,7[2,49–6,93]	0,48
Triglyceride (mmol/L)	0,66 [0,07–1,84]	0,81[0,34–3,01]	**0,012**
25(OH)D (ng/mL)	21,38 ± 10,73	16,92 ± 9063	**0,049**
PTH (pg/mL)	30,25 ± 13,98	34,05 ± 12,08	0,15
ALP (UI/L)	182,2 ± 97,9	173,1 ± 93,17	0,65
Calcium (mmol/L)	2,35 ± 0,16	2,42 ± 0,15	**0,043**
Phosphorus (mmol/L)	1,42 ± 0,23	1,39 ± 0,25	0,62

### 3.2 Vitamin D status

Hypovitaminosis D was present in 72.8 % of the GHD group cases in this study, with 25.6 % of the subjects having serum 25(OH)D values < 30 ng/mL (insufficiency) and 46.2 % having values < 20 ng/mL.

Our study revealed significant differences in the vitamin D status between the two groups (p = 0.013). In fact, we discovered that the vitamin D concentration in GHD patients was significantly higher (21,38 ng/mL ±10,73) than in healthy subjects (16,92 ng/mL± 9063) (p = 0,049) and the calcium concentration in the GHD group was significantly lower (p = 0,043) (Table 2).

In addition, statistical analysis showed a negative correlation between vitamin D and PTH (r = −0.250, p = 0.021) and a positive one between vitamin D and ALP (r = 0.443, p = 0.010) (Fig. 2).

### 3.3 Relation between polymorphisms and biological parameters

**Fig. 3:**
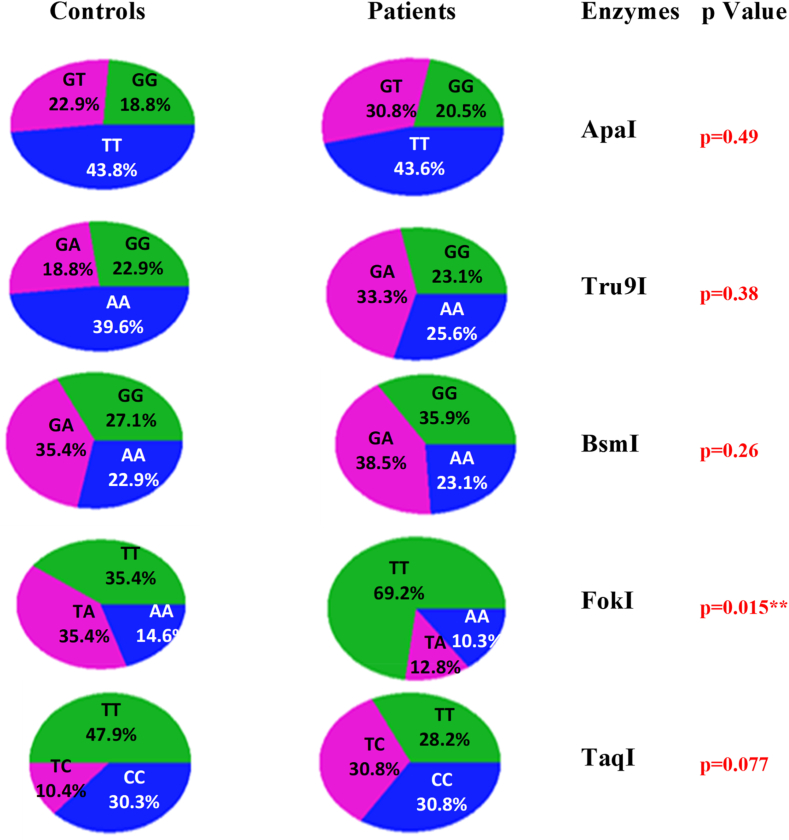
Distribution of the 5 SNP genotypes in patients and controls.

The genotypes of the *Apa*I (p = 0.49), *Tru*9I (p = 0.38), BmsI (p = 0.26), and *Taq*I (p = 0.077) polymorphisms were compared between patients and controls (Figs. S1–S5). There were no statistically significant differences between the two groups. However, a significant difference was observed for the *Fok*I polymorphism (p = 0.015) (Fig. 3).

Following stratification using biochemical parameters and polymorphisms of *Taq*I, *Apa*I, *Tru*9I, *Bsm*I and *Fok*I, no differences were found between the five SNPs and Vitamin D concentrations. However, the comparison between GHD patients and healthy subjects revealed a significant difference in genotype TT of *Taq*I (p = 0,004), both genotype GT (p = 0,031) and GG (p = 0,034) of *Apa*I and genotype TT (p = 0,049) of *Fok*I polymorphism.

**Fig. 4:**
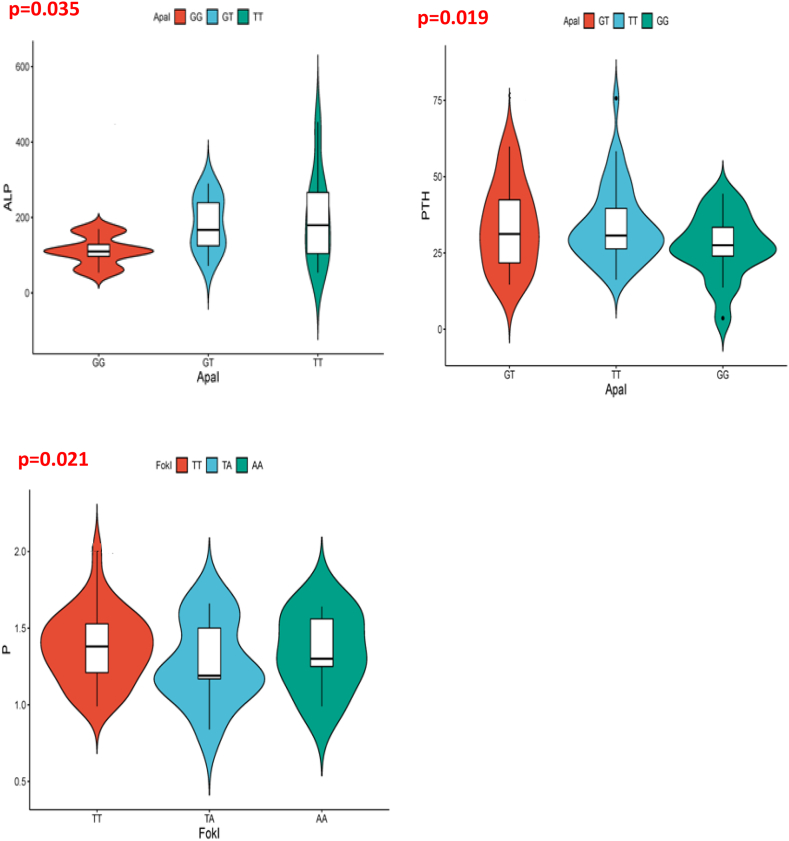
Violin plots (showing data density) and mean/standard deviation (Rectangle, vertical bar) for the association between genotypes (*Apa*I and *Fok*I) and phosphocalcic parameters (PTH, ALP, and Phosphorus (P)).

No association was seen between phosphocalcic parameters and *Taq*I, *Tru*9I, or *Bsm*I SNPs. In contrast, we found a statistically significant relationship between *Apa*I and ALP (p = 0,035), and between *Apa*I and PTH (p = 0,019) in patients with GHD. Furthermore, we discovered an association between Phosphoremia and *Fok*I polymorphism in patients (p = 0,021) (Fig. 4).

### 3.4 Haplotype analysis

**Fig. 5:**
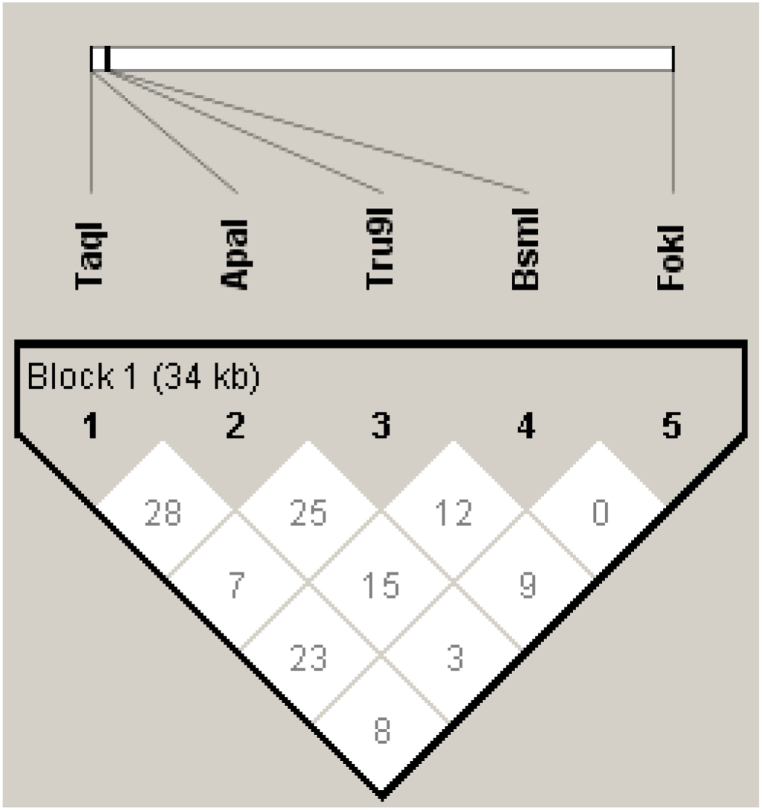
HAPLOVIEW plot of pairwise r^2^ values between a set of five simulated SNPs. The block shading shows the strength of correlation. Red and white colours indicate high and low level of LD, respectively. The r^2^ measures of LD are shown in the squares.

Haploview version 4.2 was used to estimate haplotypes after the determination of the linkage disequilibrium (LD) which is a measure of non-random association between segments of DNA (polymorphisms) at different positions on the chromosome. The LD values are relatively low (Linkage Disequilibrium <0.5), indicating that the polymorphisms are not strongly linked (Fig. 5). This weak linkage likely contributed to the identification of several haplotypes observed in this analysis.

Only one CTAGT haplotype (p = 0.0024) showed a statistically significant difference between patients and controls among the haplotypes that were obtained (Table 3).

**Table 3:** Distribution of haplotypes.

Haplotype^a^	Frequency	Patients	Controls	p
CTAAT	0,090	0,084	0,095	0,8038
TTAGT	0,088	0,081	0,094	0,7755
CTAGT	0,087	0,159	0,022	**0,0024**
TTGGT	0,072	0,088	0,057	0,4501
TGAGT	0,068	0,050	0,085	0,3789
TGGAT	0,068	0,099	0,040	0,1442
CGGAT	0,062	0,072	0,054	0,6392
TGGGT	0,049	0,057	0,041	0,6545
TTAAA	0,043	0,010	0,072	0,056
CTGGA	0,042	0,037	0,046	0,7711
TTAAT	0,034	0,019	0,048	0,314
CTAAA	0,034	0,020	0,046	0,3597
TTGAT	0,033	0,027	0,039	0,6817
CTGGT	0,033	0,045	0,022	0,4345
CTGAT	0,032	0,029	0,034	0,8581
TGAGA	0,030	0,022	0,037	0,6059
CGGAA	0,030	0,028	0,032	0,8873
TTAGA	0,024	0	0,045	0,0639
CGAGA	0,022	0,027	0,018	0,6916
TTGGA	0,017	0,007	0,026	0,3654
TGGAA	0,013	0,018	0,009	0,6211
CTAGA	0,012	0,015	0,008	0,6915
TGGGA	0,011	0,005	0,016	0,523

a Order of the SNPs: *Taq*I/*Apa*I/*Tru*9I/*Bsm*I/*Fok*I.

In the GH-deficient population, we found a statistically significant interaction between the CTAGT haplotype and PTH level (p = 0.029). After stratification with biological and anthropometric parameters (Table 4), an interaction was found between the CTAGT haplotype and calcemia (p = 0.020), phosphoremia (p = 0.020) and ALP (p = 0.020).

**Table 4:** Interaction between haplotypes and different parameters.

Variables	Haplotype CTAGT	
	Frequency	p
Vitamin D < 20 ng/mL	0,78	0,096
Vitamin D > 20 ng/mL	0,22	
Calcemia<2,2 mmol/L	0,11	**0,020**
Calcemia>2,2 mmol/L	0,89	
Phosphatemia<1,1 mmol/L	0,11	**0,020**
Phosphatemia>1,1 mmol/L	0,89	
ALP<90 UI/L	0,11	**0,020**
ALP>90 UI/L	0,89	
BMI<97^ème^ percentile	0,55	0,763
BMI>97^ème^ percentile	0,45	

### 3.5 Interaction between VDR gene polymorphisms and biological parameters

**Fig. 6:**
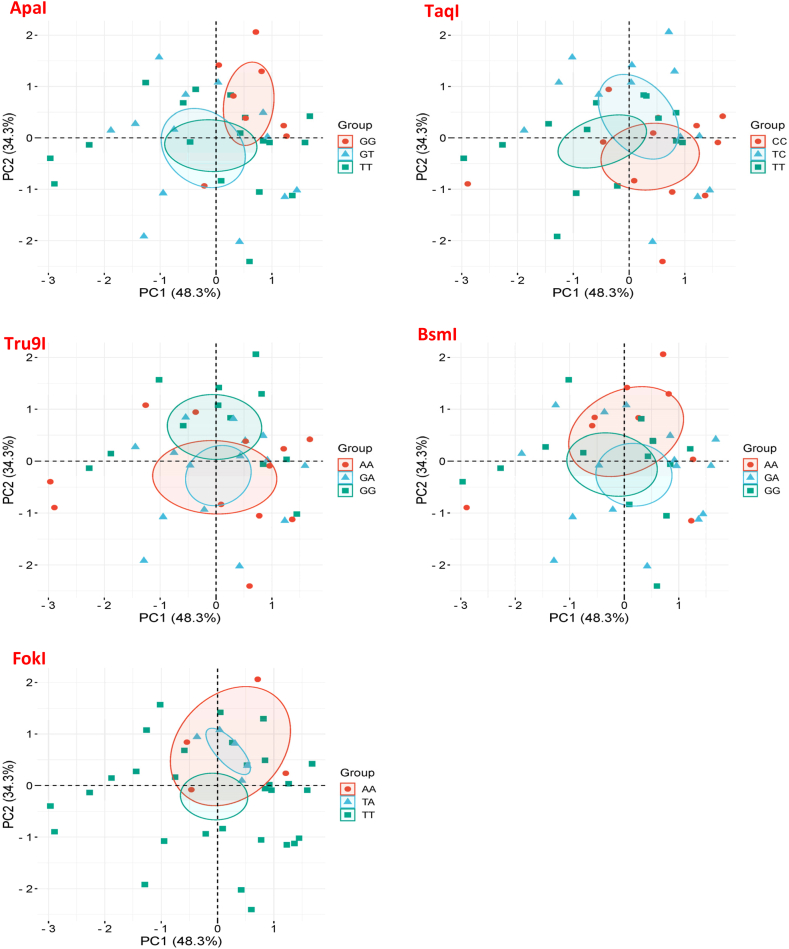
Principal Component Analysis (PCA) of different SNP genotypes according to phosphocalcic metabolism parameters (PTH, Calcium, Vitamin D, and Phosphorus).

To understand the Interaction between biological parameters and VDR gene polymorphisms, we performed a Principal Component Analysis (PCA) which is a multivariate analysis that reduces the complexity of datasets while preserving data covariance. Indeed, the three genotypes for each SNP were correlated with phosphocalcic metabolism parameters (PTH, Calcium, Vitamin D, and Phosphorus) simultaneously as shown in Fig. 6. The results showed that the genotypes GG of *Apa*I and AA of *Tru*9I were the best separated and most tightly clustered groups. The AA genotype of both *Bsm*I and the three genotypes of *Taq*I were relatively grouped with slightly overlap. The genotypes GT and TT for *Apa*I, GA and GG for both *Tru*9I and *Bsm*I, and the three genotype of *Fok*I were more strongly associated to one another indicating less variance between these groups.

The investigation of the interaction of each parameter with the 5 polymorphisms, revealed a positive association between PTH levels and *Apa*I in patients with GHD (p = 0.019). Thus, the GG genotype seemed to favour normal PTH status (p = 0.028; OR = 0.072; 95 % CI = 0.003–0.62) and low phosphorus levels (p = 0.002; OR = 0.190; 95 % CI = 0.05–0.50). Moreover, the GT genotype was associated with low ALP level (p = 0.018; OR = 0.266; 95 % CI = 0.07–0.73) and phosphorus status (p = 0.013; OR = 0.315; 95 % CI = 0.11–0.74). The *Taq*I polymorphism's TC genotype appeared to favour deficient vitamin D status (p = 0.018; OR = 0.166; 95 % CI = 0.02–0.61), low ALP status (p = 0.004; OR = 0.055; 95 % CI = 0.003–0.26), and low phosphorus levels (p = 0.001; OR = 0.173; 95 % CI = 0.05–0.45). Furthermore, the *Tru*9I polymorphism appears to be linked to poor vitamin D status and low phosphorus levels. Thus, the GA genotype favoured vitamin D deficiency (p = 0.021; OR = 0.09; 95 % CI = 0.004–0.46) and the AA genotype had a low phosphorus level (p = 0.023; OR = 0.312; 95 % CI = 0.10–0.79). For the BmsI polymorphism, the AA genotype appeared to favour low phosphorus status (p = 0.011; OR = 0.333; 95 % CI = 0.13–0.74). The TA and AA genotypes of the *Fok*I polymorphism were associated with a low ALP status (p = 0.018; OR = 0.266; 95 % CI = 0.07–0.73) and low phosphorus levels (p = 0.019; OR = 0.333 [0.12–0.79], p = 0.007; OR = 0.263; 95 % CI = 0.08–0.65). The AA genotype was also linked with vitamin D deficiency (p = 0.016; OR = 0.083; 95 % CI = 0.004–0.42) (Table 5).

**Table 5:** Interaction between Genotypes and biological parameters.

Parameters		Vitamin D status						PTH status				ALP status				P status			
		Deficient		Insufficient		Sufficient		Hypo		Normal		Hypo		Normal		Hypo		Normal	
Genotypes		p	OR [CI95 %]	p	OR [CI95 %]	p	OR [CI95 %]	p	OR [CI95 %]	p	OR [CI95 %]	p	OR [CI95 %]	p	OR [CI95 %]	p	OR [CI95 %]	p	OR [CI95 %]
***Apa*I**	**GG**	0.12	4.0000e-01 [1.0972e-01 – 1.1958e+00]	0.63	7.2368e-01 [1.9663e-01 - 3.0386e+00]	0.99	2.1617e-08 [0.3618e-41-2.3596e+52]	0.34	3 [0.38–60.64]	**0.028**	**0.072** [0.003–0.62]	0.11	0.46 [0.16–1.16]	0.21	0.47 [0.14–1.60]	**0.002**	**0.190** [0.05–0.50]	0.42	1.65 [0.50–6.51]
	**GT**	0.12	0.4 [0.11–1.19]	0.88	1.1 [0.31–4.53]	0.55	1.6 [0.29–9.74]	0.34	0.33 [0.01–2.60]	0.83	1.2 [0.15–26.66]	**0.018**	**0.266** [0.07–0.73]	0.29	1.92 [0.59–7.47]	**0.013**	**0.315** [0.11–0.74]	0.37	1.63 [0.56–5.16]
	**TT**	0.59	0.75 [0.24–2.15]	0.78	1.17 [0.35–4.06]	0.41	2 [0.39–11.16]	0.98	6.3892e-08 [1.5678e-81- 3.6323e+52]	0.98	1.7119e+07 [3.6889e-53 -5.7945e+64]	0.81	0.9 [0.35–2.23]	0.94	1.03 [0.36–2.98]	0.32	1.5 [0.68–3.44]	0.14	0.48 [0.17–1.26]
***Taq*I**	**TT**	0.59	1.33 [0.46–4.04]	0.25	0.5 [0.14–1.65]	0.72	0.75 [0.14–3.88]	0.98	6.3892e-08 [0.4325e-03 - 3.6323e+52]	0.98	1.3912e+07 [2.9998e-53 – 4.3568e+40]	0.49	0.72 [0.28–1.79]	0.89	1.07 [0.37–3.15]	0.18	0.58 [0.25–1.26]	0.40	1.5 [0.58–4.02]
	**TC**	**0.018**	**0.166 [0.02**–**0.61]**	0.44	1.8 [0.43–13.28]	0.35	2.57 [0.34–23.45]	0.99	4.2545e+07 [1.2881e-91 -5.3578 + 73]	0.99	5.0367e-09 [0.0125e-04- 1.0217e+90]	**0.004**	**0.055** [0.003–0.26]	0.06	7.02 [1.27–131.53]	**0.001**	**0.173** [0.05–0.45]	0.24	2.07 [0.64–8.07]
	**CC**	0.12	0.4 [0.11–1.19]	0.60	1.4 [0.40–5.71]	0.63	0.6 [0.72–4.11]	0.98	6.3892e-08 [1.6782e-23-3.6323e+52]	0.98	8.5371e+06 [1.8939e-53 -9.9982e+62]	0.81	1.1 [0.44–2.79]	0.05	0.35 [0.11–1.02]	0.84	0.92 [0.43–1.98]	0.06	0.38 [0.14–1.03]
***Tru*9I**	**GG**	0.25	0.5 [0.13–1.59]	0.88	0.9 [0.24–3.84]	0.48	0.5 [0.05–3.36]	0.99	1.5651e+07 [3.1156e-109 – 5.4123e+98]	0.99	2.5557e-08 [4.9512e-88 - 9.963e+107]	0.14	0.45 [0.14–1.25]	0.85	0.89 [0.27–3.22]	0.51	0.75 [0.30–1.77]	0.10	0.4 [0.13–1.22]
	**GA**	**0.021**	**0.09 [0.004**–**0.46]**	0.09	6.06 [1.03–116.04]	0.21	4.71 [0.49–106.51]	0.99	6.3892e-08 [0.9631e-79 -3.2097e+108]	0.99	6.2605e+06 [1.6444e-109 -7.8527e+94]	0.32	0.6 [0.20–1.61]	0.51	0.67 [0.21–2.28]	0.13	0.5 [0.18–1.20]	0.77	0.84 [0.28–2.64]
	**AA**	0.56	1.4 [0.44–4.73]	0.12	0.35 [0.09–1.30]	0.69	0.71 [0.12–3.90]	0.99	6.3892e-08 [0.1578e-15 -1.6444e-109]	0.99	1.1739e+07 [2.9217e-109 – 3.7539e+99]	0.14	0.45 [0.14–1.25]	0.43	1.61 [0.50–5.73]	**0.023**	**0.312** [0.10–0.79]	0.07	2.8 [0.93–9.76]
***Bsm*I**	**GG**	0.59	0.75 [0.24–2.15]	0.36	0.57 [0.16–1.99]	0.88	0.88 [0.16–4.65]	0.34	0.3 [0.01–2.60]	0.71	1.5 [0.18–31.94]	0.34	0.33 [0.01–2.60]	0.71	1.53 [0.18–31.94]	**0.011**	**0.333** [0.13–0.74]	0.26	1.8 [0.65–5.32]
	**GA**	0.12	0.4 [0.10–1.19]	0.36	1.8 [0.52–7.32]	0.55	1.6 [0.29–9.73]	0.98	6.3892e-08 [2.3579e-44 - 3.6323e+52]	0.98	1.0956e+07 [2.3863e-53 – 5.9514e+111]	0.98	6.3892e-08 [0.1473e-22 -3.6323e+52]	0.98	1.0956e+07 [2.3863e-53-4.6842e+33]	1	1 [0.47–2.11]	0.21	0.54 [0.21–1.14]
	**AA**	0.12	0.4 [0.10–1.19]	0.96	0.97 [0.27–4]	0.63	0.62 [0.07–4.11]	0.34	3 [0.38–60.64]	0.06	0.1 [0.005–0.93]	0.34	3 [0.38–60.64]	0.06	0.11 [0.005–0.93]	**0.011**	**0.333** [0.13–0.74]	0.84	1.11 [0.39–3.37]
***Fok*I**	**TT**	0.78	1.16 [0.38–3.62]	0.99	1 [0.28–3.45]	0.43	2 [0.36–12.70]	0.34	3 [0.38–60.65]	0.42	0.38 [0.01–3.19]	0.49	1.37 [0.55–3.55]	0.90	0.93 [0.31–2.68]	0.68	1.18 [0.52–2.69]	0.75	1.16 [0.43–3.12]
	**TA**	0.4.	0.62 [0.18–1.87]	0.56	0.68 [0.19–2.58]	0.34	0.4 [0.04–2.49]	0.99	2.3505e-08 [-7.7633e+90]	0.99	2.0800e+07 [9.4632e-92 -]	**0.018**	**0.266** [0.07–0.73]	0.41	1.67 [0.51–6.55]	**0.019**	**0.333** [0.12–0.79]	0.65	1.28 [0.43–4.11]
	**AA**	**0.016**	**0.083** [0.004–0.42]	0.45	2.28 [0.36–44.56]	0.84	1.33 [0.04–36.88]	0.34	0.33 [0.01–2.60]	0.52	0.46 [0.05–9.98]	**0.018**	**0.266** [0.07–0.73]	0.38	0.54 [0.14–2.32]	**0.007**	**0.263** [0.08–0.65]	0.32	0.51 [0.13–1.99]

## 4 Discussion

We conducted a case-control study that involves two groups of children: subjects with GHD diagnoses and a control group of age- and sex-matched healthy subjects.

In our study, no significant difference was found between the two groups in terms of sex (p = 0.551). In patients, the sex ratio was 1.2, with male dominance, while in controls, the sex ratio was 1. This male predominance in GH-deficient patients is attributed to the fact that boys reach pubertal growth later than girls do. This result agrees with that described in the literature [[10], [11], [12]]. In our GH-deficient population, the mean age at admission was 8.7 years, with extremes ranging from 6 to 19 years.

Most patients were between 6 and 18 years of age (78 %), which agrees with several other studies that found an average age of 12 ± 4 years at first consultation. This is still late for proper management of GH-deficient children and reveals a lack of understanding of the pathology on the part of many practitioners who have to monitor these children at an early age.

In our study, 50 % of GH-deficient children came from consanguineous marriages. This result is in line with studies emphasizing the involvement of consanguinity in the etiopathogenesis of GH deficiency [12,13]. According to the same study by Benothman W et al., carried out in Sfax, consanguinity was significantly higher in patients with combined deficiency [12].

Determination of bone age is a reliable means of diagnosing GH deficiency, given its considerable influence on bone development and differentiation of chondrocytes and osteoprogenitors [14].There was a significant delay in bone age in all patients in our study. The mean difference between the bone age and chronological age (on admission) was 5.86 years. Our results were similar to those reported by Huet et al. [10]. Other studies have reported a smaller difference, such as that of De Luca et al., who found an average of 3 years [15].

Axiological analysis (height and weight) showed that the mean weight was significantly lower in patients than in controls (p < 0.05). Similarly, the mean height was significantly diminished in GH-deficient patients (p = 0.001), in line with other studies in which a deviation below 2DS is suggestive of GHD [14,16].The delay in staturoposterior growth observed reflects both the depth of somatotropic deficiency in the study population and the delay in diagnosis.

Indeed, patients with GH deficiency experience abnormal bone remodelling, which would explain the length growth defect characteristic of these individuals; this event gradually resolves after starting of rhGH therapy. According to Durá-Travé T et al., the axiological response to the administration of recombinant human GH was quite satisfactory, since the mean value of the standard deviation (SD) was −2.83 before treatment and reached −1.29 after 48 months of treatment [17].

The current study, also, showed that BMI was comparable between patients and controls (p = 0.598). However, the frequency of malnutrition (undernutrition, overweight, and obesity) was higher in the GH-deficient patients. In addition, we observed a significant difference in protein levels between patients and controls, which was associated with a decrease in triglyceridemia (p < 0.05). These results were consistent with those reported by Metwalley et al. [18]. In fact, there is a strong relationship between GH and energy metabolism, notably between circulating levels of energy substrates such as glucose and fatty acids. Growth hormones regulate food intake via ghrelin (a potent stimulator of GH secretion) [19,20]. It also accounts for pre- and postprandial GH peaks [21].

Comparing the vitamin D status, we found a statistically significant difference between patients and controls (p = 0.013). The frequency of vitamin D deficiency was 46.2 % in deficient patients and 75 % in controls, and the frequency of vitamin D insufficiency was 25.6 % and 16.7 %, respectively. In addition, vitamin D concentrations were higher in deficient subjects (p = 0.049).

These results may be explained by the fact that GH-deficient subjects receive treatment (recombinant human GH), which affects vitamin D synthesis. Indeed, various interactions between vitamin D and the GH/IGF-1 axis have been described. For example, biological studies have shown that GH and IGF1 regulate the renal production of 1,25(OH)2D, thereby stimulating the endocrine actions of vitamin D [[22], [23], [24]].

Based on a retrospective study by Delecroix et al., the blood levels of 25(OH)D, 1,25(OH)2D, GH, and IGF-1 were measured at the time of diagnosis in 50 patients with GHD due to pituitary stem interruption syndrome (PSIS). They hypothesized a possible association between the GH/IGF-1 axis and vitamin D metabolism, given the direct proportionality between 1,25(OH)2D and 1,25(OH)2D/25(OH)D ratio values and peak GH after a pharmacological stimulation test in this population [25].

Interesting information on vitamin D status in patients with GHD before and after rhGH replacement therapy can be extrapolated from the Italian perspective study by Ciresi A et al., in which they analysed serum 25(OH)D levels at baseline and after 12 months of hormone replacement therapy in 80 prepubertal with GHD. In this study, low vitamin D values (<30 ng/mL) were found in 75 % of GH-deficient subjects. In addition, the prevalence of normal 25(OH)D levels was high after rhGH treatment for 12 months; however, no relationship was found between 25(OH)D and IGF-1, Ca, P, and PTH values in this study [23].

Nevertheless, published data are controversial because, for example, references concerning the effects of rhGH treatment on vitamin D status differ between authors who show a decrease [26] or no change [24] and those who show an increase in vitamin D during rhGH treatment [23,27,28].

The present study also showed that 22.9 % of the controls were deficient (<10 ng/mL), 68.8 % were deficient (10–30 ng/mL), and only 8.3 % had normal vitamin D concentration. This result is in line with the study by Bahlous A et al., which involved 209 healthy Tunisian subjects and found that the prevalence of hypovitaminosis D and vitamin D deficiency was 92.3 % and 47.6 %, respectively, and only 15 subjects had optimal vitamin D concentrations [29].

All these results prove the dependent relationship between vitamin D and the GH/IGF-1 axis and are also justified by the decrease in renal lα-hydroxylase activity caused by low IGF-1 levels. GH itself directly stimulates 1,25(OH)2D production [30]. In addition, GH and IGF1 appear to increase the activity of CYP27A1, a multifunctional cytochrome P450 enzyme which, among its complex functions, catalyses the 25 hydroxylation of vitamin D in hepatoblastoma cells [31].

Subjects with GHD, in addition to rhGH replacement therapy, may require vitamin D supplementation to correct the considerable risk of hypovitaminosis D. However, some studies on adults with GHD have highlighted the persistence of hypovitaminosis D despite adequate vitamin supplementation according to the main international recommendations [32].

Our results revealed that the genotypic and allelic distribution of the *Bsm*I polymorphism did not differ from those of GH-deficient patients and controls. The heterozygous GA genotype and wild-type G allele were the most frequent in both groups. This is in line with the allelic and genotypic distribution in Africa; in fact, the G allele represented 73 %, and the most frequent genotypes were GG (53.4 %) and GA (38.9 %). Several trials have demonstrated the association of the *Bsm*I polymorphism with numerous pathologies. In some cases, this SNP was associated with osteoporosis [33], pathological fractures [34], type 2 diabetes [35], prostate cancer [36], breast cancer [37], and Parkinson's disease [38].

Analysis of the *Fok*I polymorphism uncovered a predominance of the TT genotype (69.2 %) in the GH-deficient subjects (p = 0.015). The A allele was predominant in the controls, accounting for 35.9 %, while the T allele accounted for 81.4 % (p = 0.0187). In Africa, the A allele represents 18.9 %, and the T allele represents 81.1 %. The most frequent genotype was TT (65 %).

According to Divanoglou N et al., elderly residents of a homogenous rural population in central Greece exhibited a high prevalence of vitamin D deficiency, which was associated with VDR gene polymorphisms, specifically the cumulative effect of three different genotypes: *Bsm*I, *Taq*I, and *Fok*I. These findings underscore the significant correlation between VDR gene polymorphisms and serum vitamin D levels, highlighting the persistence of vitamin D deficiency in regions with abundant sunlight, such as Greece. The study also revealed a noteworthy association between low 25(OH)D3 levels and the presence of the G and C alleles of the *Bsm*I and *Taq*I polymorphisms of the VDR gene, respectively. Furthermore, it was observed that the presence of multiple unfavorable polymorphisms in the same individual had a cumulative, dose-dependent effect [39].

Other studies have concluded that VDR gene polymorphisms may be an important determinant of the amount of mRNA and receptor proteins and subsequent downstream effects mediated by vitamin D [40]. The T allele of the *Fok*I SNP leads to a longer VDR protein by directly introducing a start codon, which may influence VDR protein activity, resulting in a less efficient transcriptional activator. Furthermore, the T allele was related with higher 25(OH)D3 levels in a population-based longitudinal study [41]. Although the *Taq*I, *Apa*I, and *Bsm*I polymorphisms are situated near the 3′ end of the VDR gene, suggesting a potential correlation with gene transcription, their notable influence may extend to the regulation of mRNA stability [42,43], only a few studies have investigated the degree of genetic contribution of VDR gene polymorphisms to 25(OH)D3 and 1,25(OH)2D3 levels.

*Bsm*I and *Fok*I polymorphisms have been linked to 25(OH)D levels and VDR activity [[44], [45]]. These polymorphisms vary according to ethnicity and could explain why certain ethnic groups are more susceptible to vitamin D deficiency or vitamin D-dependent diseases than others. Hustmyer demonstrated a difference in the frequency of certain VDR polymorphisms among Caucasians, African-Americans, and Asians [46]. Finally, genetic variations in the VDR gene play an important role in an individual's sensitivity to the biological effects of vitamin D.

Conferring to a meta-analysis by Bao L et al., *Bsm*I genetic polymorphism correlates with the level of bone mineral density in children; in particular, the G allele and GG genotype are more likely to occur in children with higher bone mineral density [47]. Another study by Waziri B et al. discovered a substantial difference in PTH levels across *Bsm*I genotypes, with patients carrying the Bb genotype having a higher median PTH level. Furthermore, the Bb genotype is independently related with the probability of developing moderate to severe secondary hyperparathyroidism in individuals with end-stage renal disease [48]. Indeed, the *Bsm*I polymorphism's effect on hyperparathyroidism has been connected to the existence of the b allele. Previous research has found a clear link between b alleles and a reduction in VDR gene transcription and/or mRNA stability, influencing the regulatory activities of calcitriol on parathyroid glands [49,50]. In a study by Laaksonen M et al., the BB genotype was associated with elevated 25(OH)D concentrations and increased femoral bone mineral density, suggesting a protective role of this genotype against vitamin D deficiency [51].

According to the literature, some VDR haplotypes were associated with European or Native American ancestry of the subjects studied, but not with African ancestry, suggesting that association studies between 75 genetic variants and chronic pathologies should be adapted according to ethnicity [52,53].According to our study, only one CTAGT haplotype (*Taq*I/*Apa*I/*Tru*9I/*Bsm*I/*Fok*I) showed a statistically significant difference between the patients and controls. This suggests an association between this haplotype and GH deficiency.

After stratification by biological and anthropometric parameters, an interaction was found between the CTAGT haplotype and calcemia (p = 0.020), phosphatemia (p = 0.020), and PAL (p = 0.020). A meta-analysis identified a strong LD between SNPs located at the 3′ end of VDR [54]. According to Martin R et al., the rare AGT haplotype (*Bsm*I/*Apa*I/*Taq*I) was found to be significantly protective against diabetic nephropathy in patients with T1DM [55]. Saadi et al. showed that the TCGAT haplotype (*Fok*I/*Tru*9I/*Bsm*I/*Apa*I/*Taq*I) is associated with asthma [56].

## 5 Conclusion

This study showed that hypovitaminosis D is common in children. However, GH-deficient children have higher vitamin D levels than controls, owing to the effect of growth hormone taken as a treatment, which stimulates the endogenous synthesis of this vitamin and its endocrine actions. Thus, the pathology studied was characterized by abnormal bone remodelling leading to delayed bone maturation (bone age), hypocalcaemia, and defective staturoponderal growth. We suggest that subjects with GHD, in addition to rhGH replacement therapy, require vitamin D supplementation to correct the elevated risk of hypovitaminosis D.

In the current study, an association was highlighted between *Apa*I and PAL and PTH on the one hand, and between *Fok*I and plasma phosphorus on the other hand. This suggests the hypothetical involvement of *Apa*I and *Fok*I polymorphisms in the regulation of phosphocalcic metabolism. Our study enabled us to find a CTAGT haplotype that may be implicated in GHD by acting on GH regulation or function.

## Ethics approval and consent to participate

This study adhered to the principles outlined in the Declaration of Helsinki by the World Medical Association and received approval from the Human Ethics Committee of Bechir Hamza Children's Hospital of Tunis (Approval Number: 05/2023). All methods were executed in compliance with relevant guidelines and regulations. Informed consent to participate was obtained from all participants after receiving all the necessary information about the research. All data and identities of patients were processed with strict confidentiality.

## Consent for publication

Written informed consent for publication of their clinical details and/or clinical images was obtained from the parents.

## Funding

No funding was received for this study.

## Availability of data and materials

For Data evaluation please contact Dr. Amri Yessine at; amri.yessine@yahoo.com.

## CRediT authorship contribution statement

**Sarra Tombari:** Conceptualization, Formal analysis, Investigation, Methodology, Visualization, Writing – original draft. **Yessine Amri:** Formal analysis, Investigation, Methodology, Visualization, Writing – review & editing. **Yosra Hasni:** Formal analysis, Investigation, Writing – review & editing. **Sondess Hadj Fredj:** Investigation, Writing – review & editing. **Yesmine Salem:** Investigation. **Salima Ferchichi:** Investigation. **Leila Essaddam:** Investigation. **Taieb Messaoud:** Investigation. **Rym Dabboubi:** Conceptualization, Visualization, Writing – original draft.

## Declaration of competing interest

The authors declare that they have no known competing financial interests or personal relationships that could have appeared to influence the work reported in this paper.
