# Supplementary material for: Vitamin D status and VDR gene polymorphisms in patients with growth hormone deficiency: A case control Tunisian study
Source: Heliyon. 2024 Jul 20;10(14):e34947. doi: 10.1016/j.heliyon.2024.e34947 (PMC11325357; doi:10.1016/j.heliyon.2024.e34947)
Supplement: Multimedia component 1 [file mmc1.docx]

**Supplementary Files**

**Supplemental Table**

**Table S1: PCR primers and restriction enzymes used for SNP genotyping.**

| **Polymorphism** | **Position** | **Primer Name** | **Primer sequences** |  | **Product size (pb)** | **Annealing Tempature** | **Restriction Enzyme** | **Digestion Temperature** | **Fragment length (pb)** |  |
| --- | --- | --- | --- | --- | --- | --- | --- | --- | --- | --- |
| rs1544410  **c.1024+283 G>A** | Intron 8 | 8a F | AGTGTGCAGGCGATTCGTAG |  | 191pb | 60°C | **Bsm I** | 37°C | **GG**: 115 + 76 |  |
|  |  | 8a R | ATAGGCAGAACCATCTCTCAG |  |  |  |  |  | **AA**: 191 |  |
| rs7975232  **c.1025-49 G>T** | Intron 8 | 8b F | CAGAGCATGGACAGGGAGCAAG |  | 745pb | 65°C | **Apa I** | 37°C | **GG**: 528 + 217 |  |
|  |  | 8b R | GCAACTCCTCATGGCTGAGGTCTCA |  |  |  |  |  | **TT**: 745 |  |
| rs731236  **c.1056 T>C** | Exon 9 | 9 F | CAGAGCATGGACAGGGAGCAAG |  | 745pb | 66°C | **Taq I** | 65°C | **TT**: 494 + 251 |  |
|  |  | 9 R | GCAACTCCTCATGGCTGAGGTCTCA |  |  |  |  |  | **CC**: 293 + 251 + 201 |  |
| rs2228570  **c.2 T>A** | Exon 2 | 2 F | AGCTGGCCCTGGCACTGACTCTGGCTCT |  | 267pb | 61°C | **Fok I** | 55°C | **TT**: 265 |  |
|  |  | 2 R | ATGGAAACACCTTGCTTCTTCTCCCTC |  |  |  |  |  | **AA**: 197 + 70 |  |
| rs757343  **c.1024+443 G>A** | Intron 8 | 8c F | GCAGGGTACAAAACTTTGGAG |  | 177pb | 60.5°C | **Tru9I** | 37°C | **GG**: 117 |  |
|  |  | 8c R | CCTCATCACCGACATCATGTC |  |  |  |  |  | **AA**: 91 + 86 |  |

**Supplementary figures**

SM 1 2 3 4 5 6 7 8 9

**
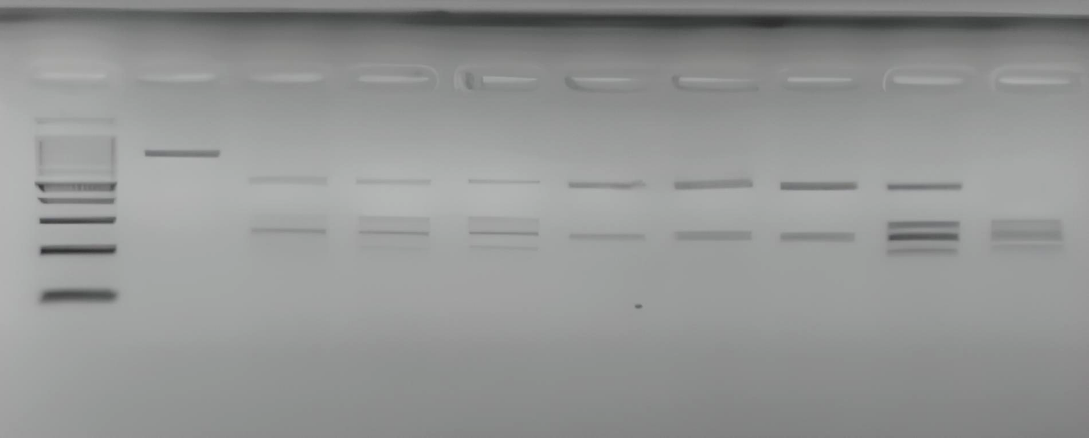
**

**745pb**

**293pb**

**251pb**

**201pb**

**494pb**

**Figure *S1*: Electrophoretic profile of enzymatic digestion products using Taq I (rs731236)**

SM: Size Marker 100pb; 1: undigested PCR product; 2, 3, 4 and 8: heterozygous profiles TC; 9: mutated homozygous profile; 5,6 et 7: normal homozygous profiles TT

SM 1 2 3 4 5 6 7 8 9


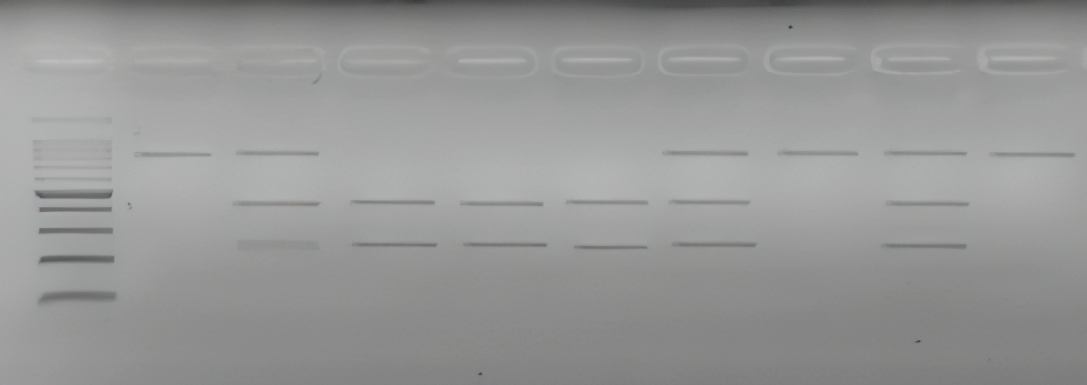


**528pb**

**217pb**

**745pb**

**Figure *S2*: Electrophoretic profile of enzymatic digestion products using Apa I (rs7975232)**

SM: Size Marker 100pb; 1: undigested PCR product; 2, 6 and 8: heterozygous profiles GT; 7 et 9: mutated homozygous profile TT; 3, 4 et 5: normal homozygous profiles GG

SM 1 2 3 4 5 6 7 8 9 10


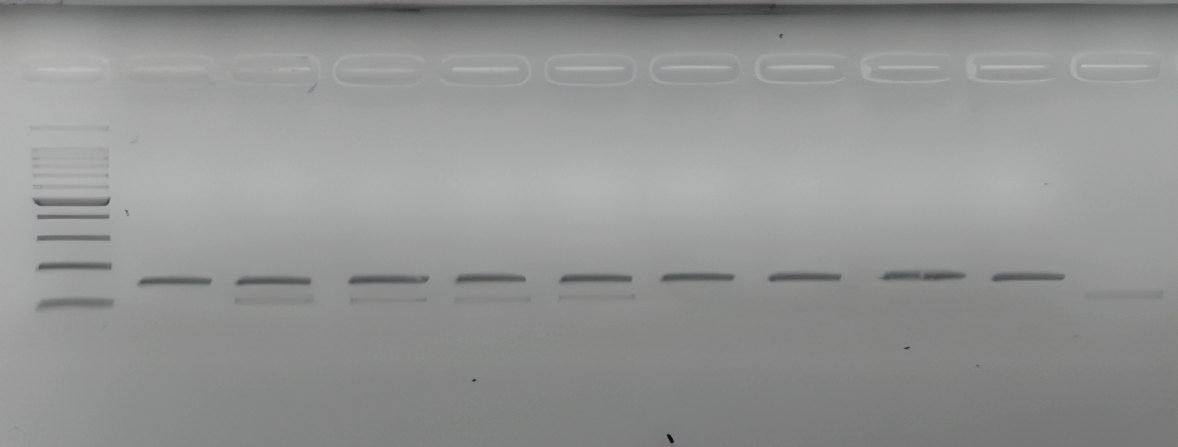


**117pb**

**91pb**

**86pb**

**117pb**

**Figure *S3*: Electrophoretic profile of enzymatic digestion products using Tru9I (rs757343)**

SM: Size Marker 100pb; 1: undigested PCR product; 2, 3, 4 and 5: heterozygous profiles GA; 10: mutated homozygous profile AA; 6, 7, 8 et 9: normal homozygous profiles GG

SM 1 2 3 4 5 6 7 8 9

**
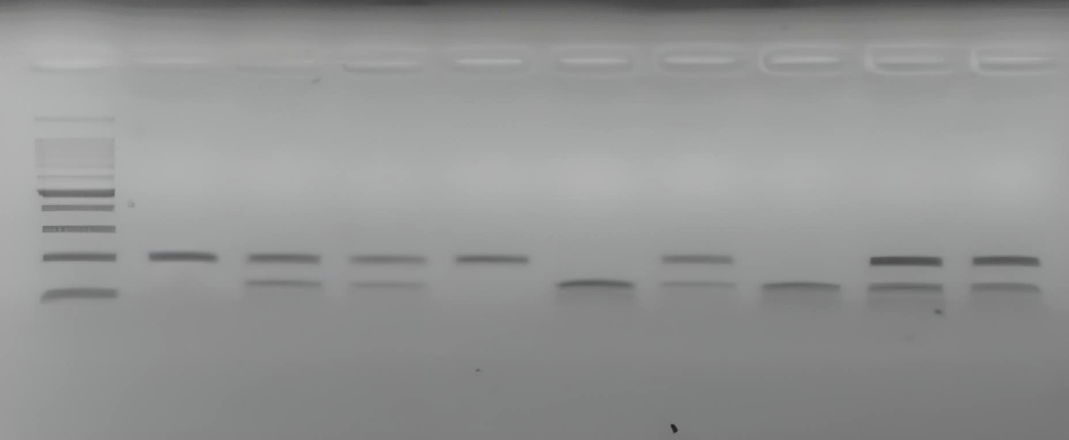
**

**115pb**

**76pb**

**191pb**

**Figure *S4*: Electrophoretic profile of enzymatic digestion products using Bsm I (rs1544410)**

SM: Size Marker 100pb; 1: undigested PCR product; 2, 3, 6, 8 and 9: heterozygous profiles TC; 4: mutated homozygous profile AA; 5 et 7: normal homozygous profiles GG


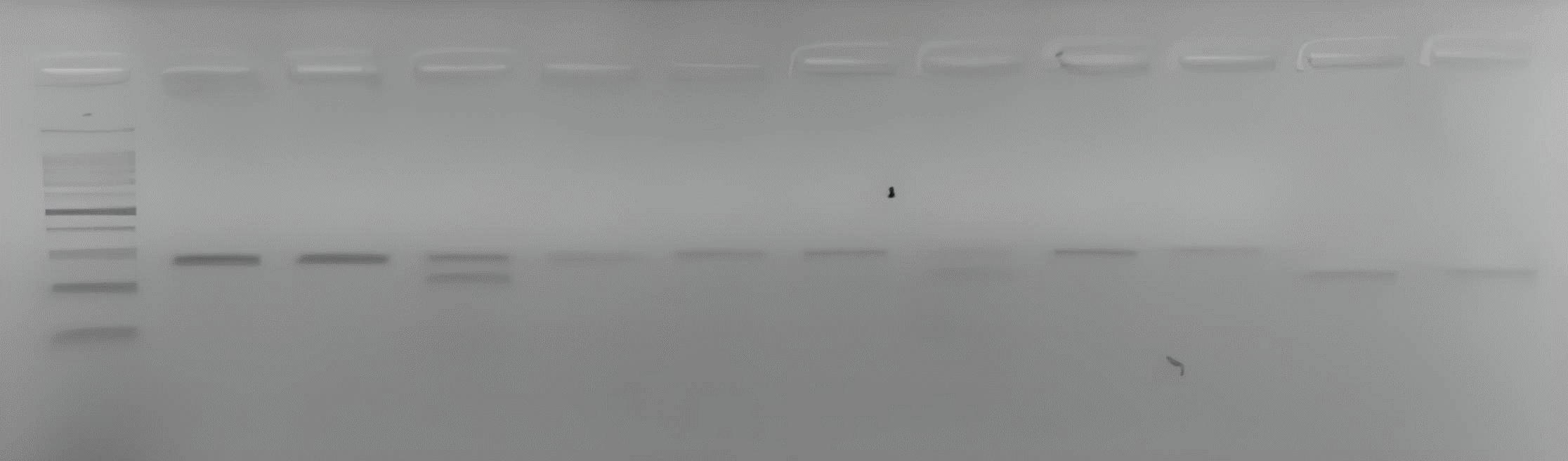


**265pb**

**197pb**

**70pb**

SM 1 2 3 4 5 6 7 8 9 10 11

**Figure *S5*: Electrophoretic profile of enzymatic digestion products using Fok I (rs2228570)**

SM: Size Marker 100pb; 1: undigested PCR product; 3 et 7: heterozygous profiles TA; 10 and 11: mutated homozygous profile AA; 2,4,5,6,8 et 9: normal homozygous profiles TT
